# Lethal Factor and Anti-Protective Antigen IgG Levels Associated with Inhalation Anthrax, Minnesota, USA

**DOI:** 10.3201/eid2002.130245

**Published:** 2014-02

**Authors:** Mark D. Sprenkle, Jayne Griffith, William Marinelli, Anne E. Boyer, Conrad P. Quinn, Nicki T. Pesik, Alex Hoffmaster, Joseph Keenan, Billie A. Juni, David D. Blaney

**Affiliations:** Hennepin County Medical Center, Minneapolis, Minnesota, USA (M.D. Sprenkle, W. Marinelli);; Minnesota Department of Health, St. Paul, Minnesota, USA (J. Griffith, B.A. Juni);; Centers for Disease Control and Prevention, Atlanta, Georgia, USA (A.E. Boyer, C.P. Quinn, N.T. Pesik, A. Hoffmaster, D.D. Blaney);; University of Minnesota, Minneapolis (J. Keenan)

**Keywords:** Inhalation anthrax, anthrax, anthrax immune globulin, critical care, anti-protective antigen, anti-PA, lethal factor, Minnesota, USA, Bacillus anthracis, zoonoses

## Abstract

*Bacillus anthracis* was identified in a 61-year-old man hospitalized in Minnesota, USA. Cooperation between the hospital and the state health agency enhanced prompt identification of the pathogen. Treatment comprising antimicrobial drugs, anthrax immune globulin, and pleural drainage led to full recovery; however, the role of passive immunization in anthrax treatment requires further evaluation.

Inhalation anthrax is a life-threatening disease caused by inhalation of *Bacillus anthracis* spores. In August 2011, the Minnesota Department of Health was contacted regarding a patient with pneumonia who had blood cultures positive for nonhemolytic gram-positive rod–shaped bacilli. We report the third naturally acquired case of inhalation anthrax reported in the United States since 1976 (*1**,**2*).

## Case Report

A 61-year-old man with a history of impaired glucose tolerance, hypertension, and chemical pneumonitis without chronic lung disease sought care at a community hospital in northwestern Minnesota after experiencing 2 days of fever, productive cough, and exertional dyspnea. During recent travel through parks in the western United States, he had been exposed to animal antlers and hides, wild bison, and donkeys.

**Figure 1:**
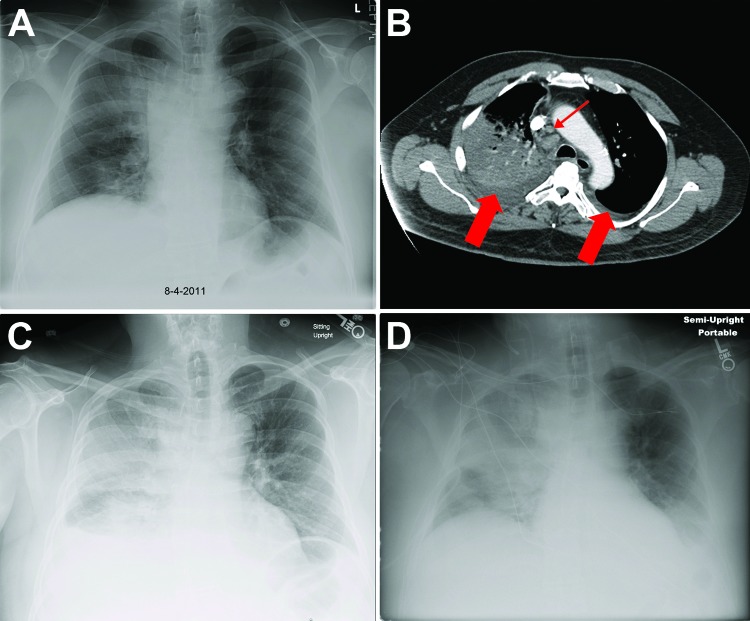
Chest x-ray and computed tomographic scan images for a patient with inhalation anthrax, Minnesota, USA. A) On hospital day 1, the x-ray image revealed a right upper lobe infiltrate and widening of the mediastinum. B) On hospital day 2, computed tomographic scan of the chest with intravenous contrast showed dense consolidation of the right upper lobe, mediastinal adenopathy (small arrow), and bilateral pleural effusions (large arrows). C) By hospital day 4, progressive infiltrates in the right lung were present. D) By day 6, an increasing left pleural effusion was evident.

On hospital admission, the patient’s temperature was 37.3°C, blood pressure 148/72, pulse 100, respiratory rate 20, and room air oxygen saturation 89% (median 95%, range 65%–100%). The right lung base had diminished breath sounds and apical crackles. Laboratory tests (Table 1) and chest imaging (Figure 1) were performed. Blood samples for culture were obtained, and ceftriaxone (2 g) and azithromycin (500 mg) (Table 2) were administered intravenously (IV) for presumed community-acquired pneumonia (CAP).

**Table 1:** Laboratory test results for patient being assessed and treated for inhalation anthrax, Minnesota, USA, 2011*

Test	Patient value, day of hospitalization†						Reference value‡
	1	3	4	5	7	9	
Hematologic							
White blood cell count (×10^3^/mm^3^)	7.6	13.9	27.4	12.0	10.7	15.6	4.0–10.0
Neutrophils (%)	73.4	82.3	81.0	70.9	75.0	75.0	34.0–70.0
Lymphocytes (%)	13.2	9.3	8.0	19.8	15.3	17.0	20.0–40.0
Monocytes (%)	12.4	8.0	9.0	7.2	5.4	3.0	4.0–10.0
Hemoglobin (g/dL)	17.0	17.0	16.6	14.5	12.8	12.7	13.1–17.5
Platelet count (×10^3^/mm^3^)	132	118	125	117	211	333	150–400
Serum chemistry							
Sodium (mEq/dL)	139	129	121	125	128	132	135–148
Potassium (mEq/dL)	3.9	4.2	3.8	3.7	3.4	4.3	3.5–5.3
Chloride (mEq/dL)	95	89	84	86	92	98	100–108
Bicarbonate (mEq/dL)	36	34	31	33	30	31	22–30
Serum urea nitrogen (mg/dL)	17	16	18	29	40	27	8–22
Creatinine (mg/dL)	1.0	0.9	0.8	1.2	1.3	0.9	0.7–1.4
Glucose (mg/dL)	248	229	158	198	186	189	70–100
Calcium (mg/dL)	8.7	8.6	8.6	7.7	7.3	7.3	8.5–10.5
Lactate (mmol/L)	ND	ND	2.2	ND	ND	ND	0.7–2.1
Albumin (g/dL)	3.6	3.0	ND	2.4	2.1	2.1	3.4–5.0
Alkaline phosphatase (U/L)	58	60	ND	63	76	80	38–126
Aspartate aminotransferase (U/L)	43	37	ND	65	44	35	5–40
Alanine aminotransferase (U/L)	92	102	ND	144	141	97	7–56
Total bilirubin (mg/dL)	0.6	0.6	ND	0.5	1.1	0.6	0.1–1.3
Troponin I (ng/mL)	ND	ND	ND	0.025	ND	ND	<0.034
Coagulation							
Prothrombin time (s)	ND	ND	11.5	11.6	ND	ND	9.0–12.5
International normalized ratio (s)	ND	ND	1.1	1.1	ND	ND	0.8–1.2
Partial thromboplastin time (s)	ND	ND	ND	27.9	ND	ND	25.0–38.0
Dimerized plasmin fragment (ng/mL)	ND	ND	ND	670	ND	ND	<229

*ND, not done. †Initial admission was to a community hospital; on day 4, the patient was transferred to a tertiary referral center. ‡Reference values during patient’s hospitalization at a tertiary referral center.

**Table 2:** Antimicrobial drugs administered to patient with inhalational anthrax diagnosed on hospital day 3, Minnesota, USA, 2011*

Antimicrobial drug	Dose	Route, frequency	Hospital and post-hospitalization days medication administered
Ceftriaxone	2.0 g	IV, every 24 h	1–4
Azithromycin	500 mg	IV, every 24 h	1–3
Ciprofloxacin	400 mg	IV, every 12 h	2–26
Meropenem	1.0 g	IV, every 8 h	3–4
Vancomycin	2.0 g	IV, once	3
Clindamycin	900 mg	IV, every 8 h	4–14
Rifampin	300 mg	Enteral, every 12 h	4–8
Meropenem	1.0 g	IV, every 8 h	8–22
Ciprofloxacin	500 mg	Oral, every 12 h	26; PH 1-35†

*IV, intravenous; hospital day, number of days in hospital including day of admission; PH, post-hospitalization days. †IV medication was discontinued and oral medication was started on day of discharge (hospital day 26) and continued for 35 additional days to complete 60 days of therapy.

On hospital day 2, the patient had increasing tachycardia and higher oxygen requirements. A computed tomographic scan of the chest revealed multiple abnormalities (Figure 1). A *Bacillus* species was isolated from blood cultures and sent to the Minnesota Department of Health Public Health Laboratory (MDHPHL) for identification, and IV ciprofloxacin was initiated. On hospital day 3, the patient’s condition continued to decline (Table 1). MDHPHL identified *B. anthracis* in the blood cultures, and meropenem and vancomycin were added to the treatment regimen. The isolate was sent to the Centers for Disease Control and Prevention (CDC, Atlanta, GA, USA).

On hospital day 4, the patient was transferred to a referral center with progressive respiratory failure requiring endotracheal intubation. He remained hemodynamically stable without need for vasopressor therapy. IV ciprofloxacin was continued, and IV rifampin and clindamycin were administered (Table 2). A chest tube was placed in the right pleural space, and 550 mL of serosanguineous fluid was drained during the initial 24 hours. Pleural fluid analysis showed a leukocyte count of 3,389 cells/mL (neutrophils 38%, lymphocytes 56%, monocytes 6%), a lactate dehydrogenase level of 352, and negative Gram stain results. On day 5, thoracentesis of the left pleural space drained 250 mL of serosanguineous fluid. Anthrax immune globulin (AIG) was requested from CDC on day 4 and administered on day 5 without adverse reaction.

The patient’s disease course was complicated by nonoliguric renal failure; serum creatinine peaked at 1.5 mg/dL. On day 8, rifampin was discontinued; meropenem, which had been discontinued on day 5, was resumed for prophylaxis against nosocomial infection and improved central nervous system coverage of *B. anthracis* infection. After stabilization, the patient was maintained on volume control ventilation: tidal volume 500 mL, positive end–expiratory pressure 10 cm H_2_O, and fraction of inspired oxygen 0.50. After day 6, renal dysfunction, hyponatremia, and thrombocytopenia gradually resolved. The patient was extubated on day 11, and the chest tube was removed on day 13; left-sided pleural effusion did not recur. He completed a 10-day course of clindamycin and a 14-day course of meropenem. Upon discharge on day 26, he was prescribed oral ciprofloxacin to complete 60 days of therapy.

MDHPHL identified the blood culture isolate as *B. anthracis* (Technical Appendix). CDC performed susceptibility testing using broth microdilution (Technical Appendix).

In compliance with the investigational new drug protocol for AIG administration, we obtained serial serum samples to assess levels of lethal factor (LF) and anti–protective antigen (PA) IgG. LF levels were determined in additional fluid from the patient’s right pleura. LF endoproteinase activity was quantified by using mass spectrometry (*3*). We used an ELISA to determine serum anti-PA IgG levels before and after AIG administration (*4*). The lower limit of quantification (LLOQ) for this assay is 3.7 µg/mL. Seroconversion was defined as a >4-fold increase over the LLOQ (*5*).

**Figure 2:**
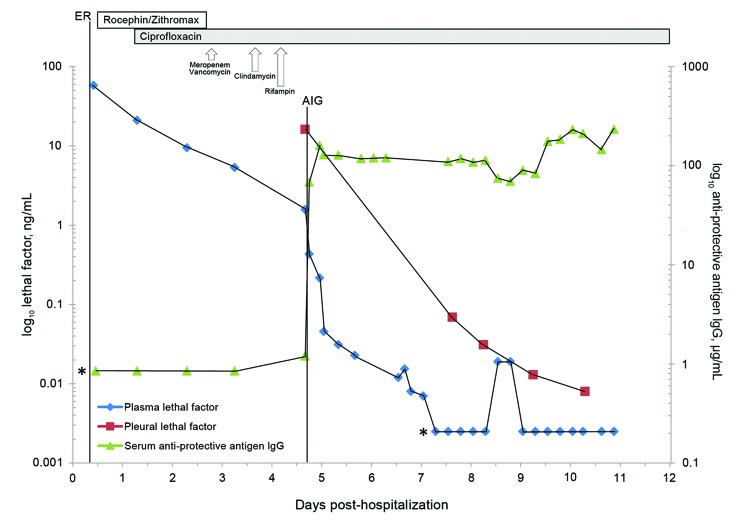
Plasma and pleural fluid lethal factor levels and anti-protective antigen IgG (AIG) levels for a patient from the time of examination in the community hospital emergency department to discharge from the tertiary referral center. Asterisks indicate that anti-protective AIG levels obtained before anthrax immune globulin administration were below the lower limit of quantification. The vertical dashed line represents the time of anthrax immunoglobulin administration. The patient’s initial plasma lethal factor level was 58.0 ng/mL and declined to 1.5 ng/mL before AIG administration. Pleural fluid LF was 16.2 ng/mL at initial drainage and declined steadily.

Antimicrobial susceptibility testing (Technical Appendix) performed on the *B. anthracis* isolate showed a MIC of penicillin of <0.015 µg/mL and MIC of ciprofloxacin of 0.12 µg/mL. The patient’s initial plasma LF level was 58.0 ng/mL, which declined to 1.5 ng/mL before AIG administration: pleural fluid LF was 16.2 ng/mL at initial drainage and declined steadily (Figure 2). Before AIG administration, no anti-PA IgG was discernable because these quantifications were below the LLOQ (Figure 2). Immediately after AIG administration, anti-PA IgG reached maximal value of 160.5 μg/mL and maintained a plateau thereafter.

## Conclusions

We describe the second US case of naturally acquired inhalation anthrax since the bioterrorism-related infections of 2001 and the third known case worldwide in which the patient received AIG (*2*,*6*). Before this case, the most recent case in the United States had occurred in Pennsylvania during 2006 (*2*). That patient had a 3-day prodromal illness, and initial plasma LF (294.3 ng/mL) and pleural fluid LF (543.2 ng/mL) levels substantially higher than those reported here. Seroconversion to anti-PA IgG occurred before AIG administration in the 2006 case, possibly because of a longer interval between symptom onset and AIG infusion (10 vs. 6 days). The 2006 patient also survived. A more recent case occurred in London during 2008 (*6*). The patient, who appears to have been in the fulminant phase of illness when tailored antimicrobial drug therapy was initiated on hospital day 4, died on day 7 despite AIG treatment (*6*).

Early recognition of inhalation anthrax is crucial to patient survival. Kuehnert et al. (*7*) proposed a scoring system derived from multivariate analysis to distinguish inhalation anthrax from CAP on the basis of clinical features at disease onset. In the case-patient described here, 3 of 5 identifying variables were present: elevated alanine aminotransferase/aspartate aminotransferase, normal leukocyte count, and tachycardia. The 2 remaining possible variables, low serum sodium level and nausea/vomiting were not present. This correlated with a sensitivity of 82% and specificity of 81% for diagnosing anthrax rather than CAP. Mediastinal widening, exhibited in the patient in this report, has also been proposed as a characteristic that can distinguish anthrax from CAP. When anthrax patients were compared with an age-, sex-, and race-matched control population, mediastinal widening occurred in 82% of anthrax patients and 8% of CAP patients (*8*).

A systematic review of inhalation anthrax cases showed improved survival if antimicrobial drugs were initiated during the prodromal rather than fulminant phase of illness: 75% of patients who survived and 10% of those who died were administered antimicrobial drugs in the prodromal phase (*9*). A new staging system for inhalation anthrax was proposed that divides the prodromal period into early and intermediate progressive stages (*10*). Early diagnosis facilitated implementation of multi–antimicrobial drug therapy during the intermediate progressive stage, which is associated with increased the survival rate (67% vs. 21%) (*9*). For the case discussed here, systems to facilitate cooperation between a community hospital and state health agency enabled definitive identification of *B. anthracis* within 24 hours of culture becoming positive, leading to specific interventions including combination antimicrobial therapy, pleural drainage, and AIG administration. 

Drainage of pleural fluid has also been associated with increased survival (83% vs. 9%) (*9*); it was performed shortly after the current patient’s illness reached the late fulminant stage. Besides elevated LF levels in pleural fluid of recent US case-patients, patients with bioterrorism-related cases had large quantities of *B. anthracis* cell wall antigens, which further supports use of drainage procedures (*11*). 

Although no clinical studies have reported on efficacy of passive immunization of humans against anthrax as treatment, mortality rates have been reduced in studies of inhalation anthrax in which animals were given polyclonal antibodies against PA (*12*). AIG administration in the current case was associated with a reduction in toxemia, although the role of passive immunization in anthrax treatment needs further evaluation.

Technical Appendix Identification and sensitivity testing of antimicrobial susceptibility of Bacillus anthracis.
