# Supplementary material for: Lethal Factor and Anti-Protective Antigen IgG Levels Associated with Inhalation Anthrax, Minnesota, USA
Source: Emerg Infect Dis. 2014 Feb;20(2):310–4. doi: 10.3201/eid2002.130245 (PMC3901492; doi:10.3201/eid2002.130245)
Supplement: Technical Appendix — Identification and sensitivity testing of antimicrobial susceptibility of Bacillus anthracis. [file 13-0245-Techapp-s1.pdf]

Article DOI: http://dx.doi.org/10.3201/eid2001.130245

Lethal Factor and Anti-Protective Antigen IgG Levels Associated with Inhalation Anthrax, Minnesota, USA

Technical Appendix: Identification and Complete Sensitivity Testing of *B. anthracis* Isolate

Bacterial Identification

MDHPHL identified the blood culture isolate as *Bacillus anthracis* through 2 processes. First, nucleic acid amplification was positive for 3 markers (BA1, BA2, BA3) specific for *B. anthracis*. Second, identification was confirmed by demonstrating capsule production and gamma phage assay.

Complete Sensitivity Testing

Antimicrobial susceptibility testing performed on the *B. anthracis* isolate revealed MICs to penicillin ≤0.015 µg/mL, doxycycline ≤0.015 µg/mL, levofloxacin = 0.12 µg/mL, tetracycline = 0.06 µg/mL, ciprofloxacin = 0.12 µg/mL, clindamycin ≤0.25 µg/mL, and meropenem = 0.03 µg/mL.
